# Mechanical Model for Super-Anisotropic Swelling of the Multi-Cylindrical PDGI/PAAm Gels

**DOI:** 10.3390/polym15071624

**Published:** 2023-03-24

**Authors:** Tasuku Nakajima, Kei Mito, Jian Ping Gong

**Affiliations:** 1 Faculty of Advanced Life Science, Hokkaido University, N21W11, Kita-ku, Sapporo 001-0021, Japan; 2 Institute for Chemical Reaction Design and Discovery (WPI-ICReDD), Hokkaido University, N21W10, Kita-ku, Sapporo 001-0021, Japan; 3 Graduate School of Life Science, Hokkaido University, N10W8, Kita-ku, Sapporo 060-0810, Japan

**Keywords:** gel, anisotropic swelling, lipid bilayer, X-ray diffraction, buckling, actuator

## Abstract

MC-PDGI/PAAm gels are cylindrical composite gels containing poly(dodecyl glyceryl itaconate) (PDGI) as a polymerized lipid oriented in a multilayer tubular shape within a polyacrylamide (PAAm) network. The most unique feature of the MC-PDGI/PAAm gel is its super-anisotropic swelling, wherein the diameter of the gel increases, but the length decreases with an increase in the volume of the gel. Through swelling and small-angle X-ray diffraction experiments, we investigated the effects of PDGI lipid bilayers and polymer network on the swelling of the MC-PDGI/PAAm gel, which suggests that the swelling anisotropy of the MC-PDGI/PAAm gel is dominated by the elasticity of the PDGI bilayers. Furthermore, we investigated the equation of state of the gel that roughly reproduced the experimental swelling results. These findings are crucial for realizing the controlled super-anisotropic swelling of MC-PDGI/PAAm gels and their applications as anisotropic actuation devices.

## 1. Introduction

The swelling extent of a polymer gel changes in response to its chemical and mechanical environments. This environment-responsive swelling of a gel enables its application in stimuli-responsive actuators and sensors [1,2]. While the most gels show isotropic swelling owing to their isotropic structure, gels exhibiting anisotropic swelling ability have attracted significant interest as anisotropic actuation devices [3]. For application as anisotropic actuators, anisotropic gels should exhibit reversible anisotropic swelling ability with large swelling anisotropy. Although anisotropic swelling gels have been synthesized using various methods [3,4,5,6,7,8,9], from the viewpoint of reversible and highly anisotropic swelling, the introduction of ordered two-dimensional sheet-like domains into gels is the most successful method that aligns with the above requirement [10,11,12,13,14].

An anisotropic swelling gel with sheet-like domains, monodomain PDGI/PAAm hydrogels consisting of a polyacrylamide (PAAm) network as a gel matrix, and periodically stacked monodomain poly(dodecyl glycidyl itaconate) (PDGI) lamellar lipid bilayers has been reported [14,15]. The PDGI/PAAm gel is synthesized from a precursor solution containing a polymer network precursor and dodecyl glycidyl itaconate (DGI) as a polymerizable lipid with trace co-surfactants that form a polydomain periodic lamellar structure in the solution [16]. The precursor solution was poured into the mold with strong mechanical shear such that the DGI lipid bilayers were uniaxially oriented along the walls of the mold to form a monodomain structure of the order of centimeters [14]. After polymerization of the precursors, the monodomain lipid bilayers of the polymerized DGI were fixed in PDGI/PAAm gels. As the PDGI layers and PAAm networks adhere via hydrogen bonding [17], the deformations of the PAAm network and PDGI bilayers are well coupled. Owing to their periodic and well-oriented lamellar structure, PDGI/PAAm gels exhibit unique properties such as anisotropic swelling [14,18], anisotropic deformation [19], stimuli-responsive structure color [20,21], low-dimensional diffusion [18,22], and high mechanical toughness [23].

**Figure 1:**
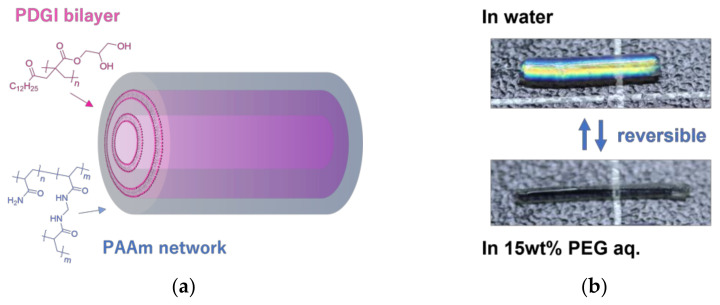
Introduction of MC-PDGI/PAAm gels, which are cylindrical composite gels consisting of the multitubular poly(dodecyl glycidyl itaconate) (PDGI) bilayers and polyacrylamide (PAAm) gel matrix: (**a**) Schematic of an MC-PDGI/PAAm gel, where thousands of PDGI lipid bilayers are periodically stacked in PAAm gel matrix. (**b**) MC-PDGI/PAAm gel exhibiting reversible super-anisotropic swelling, where the diameter increases but length decreases with the volume increase and excellent reversibility.

The original monodomain PDGI/PAAm gels synthesized using a rectangular mold had planar PDGI lamellar bilayers inside the rectangular gels [14]. When the rectangular PDGI/PAAm gels were subjected to swell/de-swell, they underwent major out-of-plane deformation, but they did not exhibit significant in-plane deformation. The nearly perfect one-dimensional swelling/de-swelling of the rectangular PDGI/PAAm gels corresponds to their remarkable swelling anisotropy. Cylindrical PDGI/PAAm gels with multitubular PDGI bilayers (MC-PDGI/PAAm gels) were fabricated using tubular molds (Figure 1a) [18]. Interestingly, the MC-PDGI/PAAm gels exhibited an even greater swelling anisotropy upon swelling. When the as-prepared MC-PDGI/PAAm gel swells, it expands along the radial direction but contracts along the longitudinal direction, despite an increase in volume (Figure 1b). Moreover, this unique swelling behavior of the MC-PDGI/PAAm gel was reversible and could be repeated multiple times.

The unique super-anisotropic swelling of the PDGI/PAAm gels was attributed to the coupling of the rubber elasticity of the PAAm network and membrane elasticity of the PDGI lipid bilayers. The PAAm network shows (isotropic) rubber elasticity and prefers isotropic deformation; however, the lipid bilayers prefer deformation without an area change. The anisotropic deformation of PDGI/PAAm gels can be understood by considering the balance of these two different elasticities. Based on this idea, the mechanical models that describe the anisotropic deformation of rectangular PDGI/PAAm gels have been proposed [19,24]. However, these models cannot be directly applied to the deformation of MC-PDGI/PAAm gels because of the complex relationship of the deformations of the gel and bilayer. For rectangular gels, in-plane deformation accompanies a bilayer area change, whereas out-of-plane deformation does not. According to the mechanical model for a rectangular gel, it swells only along the out-of-plane direction because the bilayer area change accompanied by in-plane deformation is strongly unfavorable. In conclusion, the precise mechanical modeling of the PDGI bilayer is not necessary because the bilayers act as rigid membranes that prohibit in-plane deformation. However, in MC-PDGI/PAAm gels, both radial and longitudinal deformations inevitably accompany the area change in the bilayer. Moreover, a previous study reported that the anisotropic swelling behavior of MC-PDGI/PAAm gels change drastically at a certain swelling ratio [18], probably due to the membrane buckling. Therefore, in order to accurately express the extremely anisotropic swelling of MC-PDGI/PAAm gels with a mechanical model, it is imperative to investigate the structure of the PDGI bilayers in the gels swollen at various extent and to develop a precise formula that explains elasticity of the PDGI bilayers including buckling.

The purposes of this study were to investigate the bilayer structure of MC-PDGI/PAAm gels at different swelling extents and to establish a mechanical model that reproduces their super-anisotropic swelling behavior. We report (1) effect of the cross-linking density of the polymer network on the anisotropic swelling of the MC-PDGI/PAAm gels, (2) evaluation of the PDGI bilayer structure in the MC-PDGI/PAAm gel by small-angle X-ray diffraction, and (3) construction of a deformation model based on these results.

## 2. Materials and Methods

### 2.1. Materials

Itaconic acid anhydride, dodecanol, glycidole, pyridinium *p*–toluenesulfonate, acrylamide (AAm), *N*,*N*’-methylenebisacrylamide (MBAA), Irgacure 2959, sodium dodecyl sulfate (SDS), and polyethylene glycol (PEG; number-average molecular weight Mn: 21,170) were used as received. List of chemicals with their supplier and reported assay are shown in Table S1.

### 2.2. Synthesis of Dodecyl Glyceryl Itaconate

Dodecyl glyceryl itaconate (DGI) was synthesized following the previous report [21]. First, dodecyl itaconate (DI) was synthesized as a precursor of DGI. Itaconic acid anhydride (50.1 g, 0.44 mol) was mixed with dodecanol (80.0 g, 0.43 mol) under an argon atmosphere in a round flask. The flask with the mixture was then heated to 110 °C by an oil bath for 50 min to synthesize DI. A total of 100 mL hexane was added to the flask at 70 °C to obtain crude DI precipitate. The obtained DI was recrystallized twice with ethanol. Subsequently, recrystallized DI (20 g, 0.067 mol), glycidole (15 g, 0.20 mol), and pyridinium *p*-toluenesulfonate (40 mg, 0.16 mmol) as a catalyst were dissolved in 20 mL toluene under argon atmosphere in a round flask. The flask with the mixture was then heated to 100 °C by an oil bath for 5 h to synthesize DGI. After removal of toluene by a vacuum concentrator, DGI was purified by column chromatography on silica gel (eluent: hexane/ethyl acetate = 1:1). The obtained DGI was further purified by recrystallization with acetone/hexane = 1:1 two times.

### 2.3. Gel Synthesis

The MC-PDGI/PAAm gels were synthesized as previously reported [14]. Briefly, DGI (0.1 M), SDS (0.025 mM), AAm (2 M), MBAA (0.1–0.5 mol% (to AAm)), and Irgacure 2959 (2 mM) were dissolved in pure water. The precursor solutions were kept at 55 °C for approximately 5 h. During the treatment, we shook the precursor solutions very gently several times every hour to form a stable DGI lamellar bilayers. Formation of the bilayers was confirmed by stable bright structural color of the solutions. These solutions were then moved to an argon blanket and suctioned into a polyethylene tube (inner diameter: 1.0 mm, length: approximately 30 cm) using a 10 mL syringe with the shear rate of 1300 s^–1^ to induce macro-scale orientation of the DGI multi-lamellar bilayer along the wall of the tube. Subsequently, the DGI and AAm were polymerized by 365 nm UV light (intensity: 4 mW/cm^2^) irradiation in the tube for 8 h at 50 °C. The prepared MC-PDGI/PAAm gels were removed from the tubes. String and rectangular PAAm gels without PDGI bilayers were also prepared from the precursor aqueous solutions containing AAm (2 M), MBAA (0.1–0.5 mol% (to monomer)), and Irgacure 2959 (2 mM). The as-prepared MC-PDGI/PAAm gels and PAAm gels were used as the reference states. The as-prepared MC-PDGI/PAAm and PAAm gels were cut into several pieces with a longitudinal length of approximately 1 cm for the following experiments.

### 2.4. Swelling Ratio Measurements

Optical images of cut samples were obtained using an optical microscope. The initial sample length *L*_0_ and diameter *D*_0_ were determined by analyzing taken photos using ImageJ software. Three repeated measurements of the length and diameter were performed on the same piece, and the average values were used as the data. Subsequently, the cut samples were immersed in pure water or 2–50 wt% PEG aqueous solutions for at least 3 d until swelling equilibrium was reached. The sample length *L* and diameter *D* after swelling were determined using the same image analysis method.

For each independent experiment, we synthesized and measured three pieces of a sample. For swelling ratios in water, we performed two independent experiments (thus, the sample size was 2 × 3 = 6). For swelling ratios in PEG solutions, we performed one independent experiment (the sample size was 3).

### 2.5. Small Angle X-ray Diffraction (SAXD) Measurements

Small angle X-ray diffraction (SAXD) measurements were performed at the BL05XU of SPring-8 (Japan Synchrotron Radiation Research Institute (JASRI), Japan). The MC-PDGI/PAAm gels swollen in pure water or PEG aqueous solutions were sealed with two Kapton films to prevent drying during the measurement. The samples were irradiated with X-rays (wavelength, λ = 0.1 nm) at room temperature for 20 s, and the diffraction pattern was recorded using a detector (camera length: 2.249 or 3.797 m). After background substitution, the obtained 2-D diffraction images were integrated into the 1-D scattering profile with an azimuth angle range of 70°–110° using the software Fit2D, where a 0° azimuth angle corresponds to the longitudinal direction of the sample. In the 1-D profiles, the X-ray intensity *I* is plotted against the scattering vector q=4πsinθ/λ. The peaks in the 1-D profiles were fitted by the Lorentzian function to estimate their position and full width at half maximum (FWHM). Each data point is from a single measurement.

## 3. Results

### 3.1. Anisotropic Swelling

**Figure 2:**
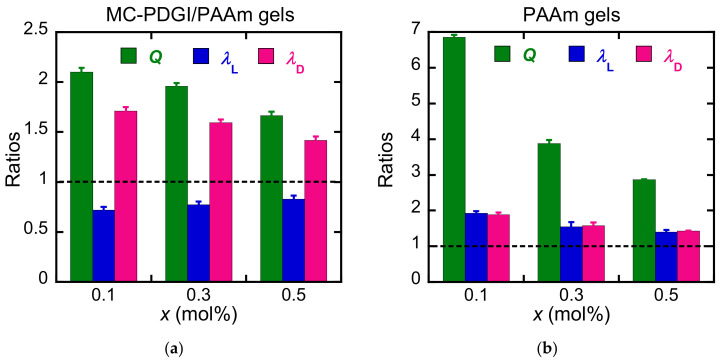
Longitudinal deformation ratio $\lambda_{L}$, radial deformation ratio $\lambda_{D}$, and volume swelling ratio *Q*, of (**a**) MC-PDGI/PAAm(*x*) gels and (**b**) PAAm(*x*) gels immersed in pure water. Note that the scale of the *y*-axis is different for each graph. The error bars represent mean ± SD (*n* = 6).

First, we investigated the effect of the elasticity of the PAAm polymer network on the anisotropic swelling of the MC-PDGI/PAAm gels. We synthesized a series of MC-PDGI/PAAm gels with different polymer network cross-linking densities controlled by MBAA (cross-linker) concentration. The sample code “MC-PDGI/PAAm(*x*) gels” was used to describe each sample, where *x* = 0.1, 0.3, and 0.5 is the feed MBAA concentration (mol%) in the gel precursor solution. String PAAm(*x*) gels without bilayers were also synthesized as the control samples. The equilibrium swelling behavior of the gels immersed in pure water was first investigated. Figure 2 shows the longitudinal deformation ratio $\lambda_{L}=L/L_{0}$, radial deformation ratio $\lambda_{D}=D/D_{0}$, and volume swelling ratio $Q=LD^{2}/L_{0}D_{0}{}^{2}$ of the MC-PDGI/PAAm(*x*) and PAAm(*x*) gels immersed in pure water. *L* and *D* are the length and diameter of the sample after swelling, respectively. $L_{0}$ and $D_{0}$ are the length and diameter of the sample in their as-prepared (reference) state, respectively. The PAAm gels generally showed a larger swelling ratio *Q* than the corresponding MC-PDGI/PAAm gels because the membrane elasticity of the PDGI bilayers suppressed the swelling of the MC-PDGI/PAAm gels. In addition, *Q* of both the MC-PDGI/PAAm and PAAm gels generally decreased with the increasing cross-linker concentration *x*. This is the common behavior of gels in their good solvents, and it is implied that dense cross-linking leads to an increase in the elastic energy density of a polymer network, which works to suppress its swelling [25]. Regarding swelling anisotropy, PAAm gels showed isotropic swelling behavior ($\lambda_{D}=\lambda_{L}$). In contrast, all MC-PDGI/PAAm gels showed $\lambda_{D}>1$ but $\lambda_{L}<1$ regardless of the cross-linking density *x*. This implies that as the gel swells (volume increases), the diameter of the gel increases, but the length of the gel decreases.

**Figure 3:**
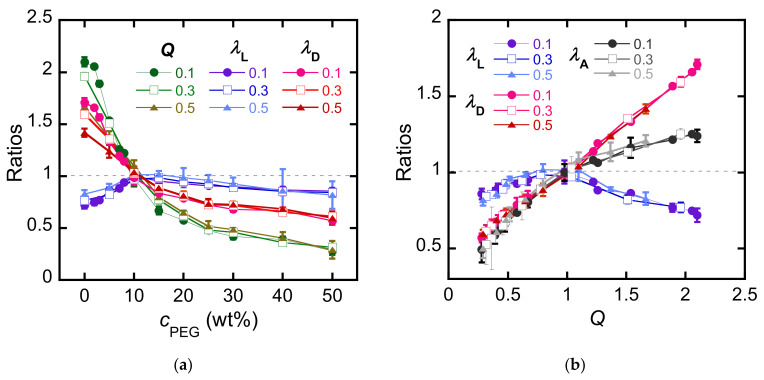
(**a**) $\lambda_{L}$, $\lambda_{D}$, and *Q* of MC-PDGI/PAAm(*x*) gels in polyethylene glycol (PEG) aqueous solutions as functions of *c*_PEG_. (**b**) $\lambda_{L}$, $\lambda_{D}$, and $\lambda_{A}$ of the MC-PDGI/PAAm(*x*) gels as functions of *Q*. The error bars represent mean ± SD (*n* = 3).

Subsequently, the MC-PDGI/PAAm gels were immersed in PEG aqueous solutions of various concentrations (*c*_PEG_) to control the volume swelling ratio of the gels by controlling the osmotic pressure of the external solutions [26]. Figure 3a shows $\lambda_{L}$, $\lambda_{D}$, and *Q* values of the MC-PDGI/PAAm(*x*) gels as functions of the outside PEG concentration *c*_PEG_; *Q* of the MC-PDGI/PAAm gels decreases with *c*_PEG_ regardless of *x*; $\lambda_{D}$ of the gels also decreases monotonically with an increase in *c*_PEG_. Moreover, $\lambda_{L}$ of the gels showed a non-monotonic dependence on *c*_PEG_; it showed a positive dependence on *c*_PEG_ at small *c*_PEG_ but a negative dependence on *c*_PEG_ at large *c*_PEG_; it showed the maximal value at a *c*_PEG_ value of approximately 10 wt%. Thereafter, we replotted the deformation ratios of the MC-PDGI/PAAm gels as functions of *Q*. In addition to the 1-D deformation ratios, we analyzed the surface area deformation ratio $\lambda_{A}=LD/L_{0}D_{0}$. As the PDGI bilayer forms a multitubular bilayer structure in the gel, $\lambda_{A}$ is also the “apparent” areal deformation ratio of the PDGI bilayers. Figure 3b shows $\lambda_{L}$, $\lambda_{D}$, and $\lambda_{A}$ as functions of *Q*. Interestingly, all the data collapse in one master curve regardless of *x*; the formation of the master curves indicates that once the volume swelling ratio *Q* of the MC-PDGI/PAAm gel is given, its swelling anisotropy (a pair of $\lambda_{L}$ and $\lambda_{D}$ at a given *Q*) is determined almost independently of the cross-linking density *x*. This strongly suggests that the swelling anisotropy of the MC-PDGI/PAAm gels is dominated by the PDGI bilayers, whereas the cross-linking density of the polymer network affects the absolute volume swelling ratio *Q*, especially at small *c*_PEG_. Note that we previously reported isotropic swelling of the string PAAm(0.1) gel in the PEG aqueous solutions [18]. $\lambda_{L}$ and $\lambda_{D}$ of the PAAm(0.1) as functions of *Q*, reproduced from [18], are shown in Figure S3.

Subsequently, the shapes of the master curves obtained were examined. At Q>1, $\lambda_{D}$ of the MC-PDGI/PAAm gel increases, but $\lambda_{L}$ decreases with an increase in *Q*, corresponding to extreme anisotropic swelling. At this “swelling” regime, the diameter and length of the gel change oppositely upon swelling/de-swelling. Additionally, in this regime, the bilayer expanded compared with the initial state ($\lambda_{A}>1$). However, at Q<1, both $\lambda_{D}$ and $\lambda_{L}$ of the gel decreased with a decrease in *Q*. At this “de-swelling” regime, although the gel still exhibits large swelling anisotropy ($\lambda_{L}>\lambda_{D}$), the anisotropy is less remarkable than that in the swelling regime. The bilayers were compressed ($\lambda_{A}<1$) in this de-swelling regime. Generally, a compressed elastic membrane buckles at a certain threshold stress/strain [27,28]. As the PDGI bilayers are very thin (approximately 5 nm) with a small bending modulus, the threshold stress/strain of the PDGI bilayers for buckling might be relatively low. Therefore, the PDGI bilayers are expected to buckle with an extremely small compression. Consequently, the transition of the swelling and de-swelling regimes may originate from the buckling of the compressed PDGI bilayers at $\lambda_{A}<1$.

### 3.2. Structural Analysis

**Figure 4:**
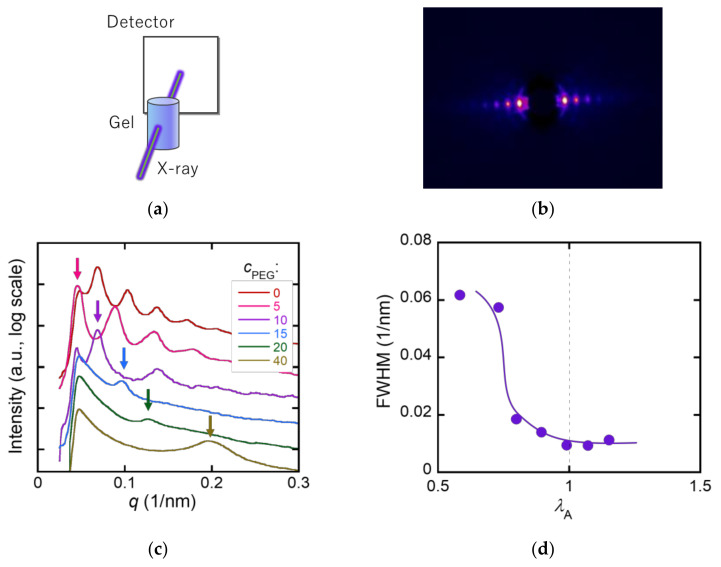
(**a**) Experimental setup for the small angle X-ray diffraction (SAXD) measurement. (**b**) 2-D X-ray diffraction image of the MC-PDGI/PAAm(0.1) gel swollen in pure water. (**c**) 1-D diffraction profile of the MC-PDGI/PAAm(0.1) gel immersed in various PEG aqueous solutions. The arrows in the figure correspond to the position of the first-order peak. For *c*_PEG_ = 0, the first-order peak was out of the *q* range. The graphs are offset vertically for clarity. (**d**) Full width at half maximum (FWHM) of the first-order peak of the 1-D profile as a function of $\lambda_{A}$.

Determining the structure of the PDGI bilayers inside the MC-PDGI/PAAm gels is important to understand the anisotropic deformation behavior of the gels. However, as the PDGI bilayers are very thin and thermally fluctuating in the gels, direct observation of the PDGI bilayers is challenging. Therefore, we adopted SAXD to obtain indirect evidence of the bilayer structure in the gels. X-rays were irradiated from the side walls of the MC-PDGI/PAAm(0.1) gels, as shown in Figure 4a. For example, Figure 4b shows a 2-D X-ray diffraction image of the MC-PDGI/PAAm(0.1) gel swollen in water. Clear periodic diffraction patterns appeared at azimuth angles of 90° and 270°, corresponding to the monodomain lamellar structure of the PDGI bilayers in the gel [15]. The 2-D images of the gels immersed in PEG solutions are shown in Figure S1; these 2-D images were processed into 1-D profiles in the azimuth angle range of 70°–110°. Figure 4c shows the selected 1-D X-ray diffraction profiles of the MC-PDGI/PAAm(0.1) gels immersed in various PEG aqueous solutions. The diffraction peaks shifted to the high *q* regime with an increase in *c*_PEG_ owing to the de-swelling-induced reduction in the spacing of the periodic PDGI bilayers. Herein, we focus on the FWHM of the first-order peaks to evaluate the buckling of bilayers, and the results are summarized in Figure 4d. At $\lambda_{A}>1$, where the PDGI bilayers were expanded, the FWHM of the first-order peak of the MC-PDGI/PAAm(0.1) gel was small. Notably, the FWHM could not be determined when *Q* was large because the first-order peak did not fall within the observation window owing to the large bilayer spacing. Therefore, for *Q* > 1, an additional SAXD experiment, with a longer camera length, was performed. The first-order peak was obtained in all cases, and the FWHM in this regime was nearly constant regardless of *Q* (Figure S2). In contrast to the swelling regime, at $\lambda_{A}<1$, where the bilayers were compressed, the FWHM significantly increased with a decrease in $\lambda_{A}$. The increase in the FWHM at $\lambda_{A}<1$ can be interpreted as an increase in the buckling amplitude of the PDGI bilayers with de-swelling. When the apparent area of the bilayers decreased owing to de-swelling, the bilayers were compressed and began to buckle. With the progression of de-swelling (decrease in $\lambda_{A}$), the amplitude of bilayer buckling should increase further; this assumption is consistent with our experimental observation of an increase in the FWHM of the MC-PDGI/PAAm gels with a decrease in $\lambda_{A}$.

**Figure 5:**
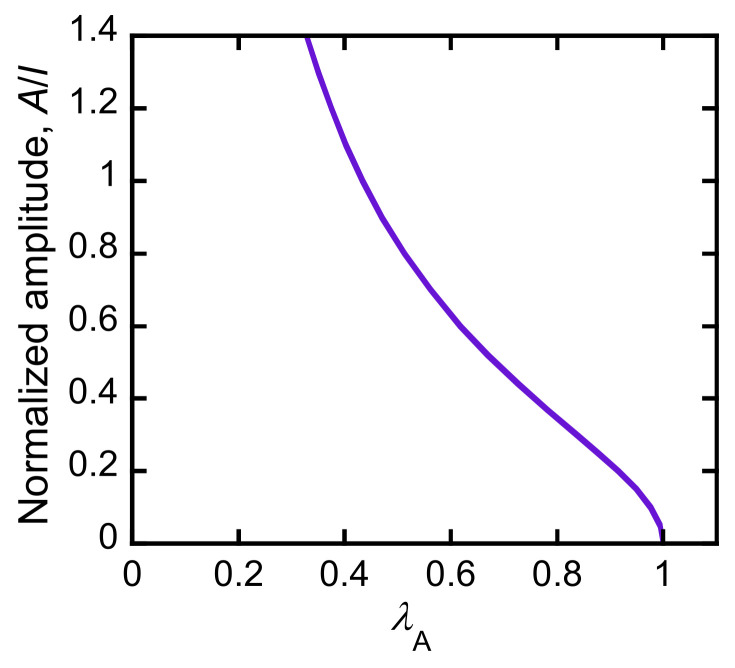
Estimated normalized amplitude of the sinusoidally buckled bilayers as a function of $\lambda_{A}$.

In principle, the relationship between the buckling amplitude and $\lambda_{A}$ can be understood using a mechanical model of buckling. To roughly estimate the relationship between them, we adopted the simple buckling model of a thin film laminated on a compliant substrate proposed by Chung et al. [29]. They assumed that an elastic thin film adhered to a soft substrate was uniaxially compressed, which subsequently formed a sinusoidal shape. The shape of the buckled film is thus characterized by two factors: buckling wavelength λ and buckling amplitude *A*. Although some of the conditions of this model differ from our system (*ex.* they assumed a non-fluidic membrane, but a PDGI lipid bilayer might be considered fluidic), we considered this model to be roughly applicable to our system. According to the model, the buckling wavelength l of the film on the substrate is given by

(1) $$l=2\pi h{\left(\frac{{\bar{E}}_{\mathrm{film}}}{3{\bar{E}}_{\mathrm{substrate}}}\right)}^{\frac{1}{3}},$$

where *h* is the thickness of the thin film (PDGI bilayers), and ${\bar{E}}_{\mathrm{film}}$ and ${\bar{E}}_{\mathrm{substrate}}$ are the plane-strain moduli of the thin film and soft substrate (PAAm network), respectively. According to this equation, the buckling wavelength l of a thin film on a substrate is determined by the modulus ratio of the two components and film thickness and is independent of the degree of compression. This implies that l remains constant, but the buckling amplitude *A* increases as the film compression proceeds. The numerical analysis gives an approximate relationship between $\lambda_{A}$, which corresponds to the degree of compression of the PDGI bilayers, and normalized buckling amplitude *A*/*l*. Figure 5 shows the estimated *A*/*l* values as a function of $\lambda_{A}$. For the estimation, *A*/*l* of a sinusoidal curve with a given wavelength *l* as a function of its arc length was calculated with numerical analysis. As shown in the figure, the normalized buckling amplitude of the PDGI bilayers increased with a decrease in $\lambda_{A}$, which is consistent with the increase in the FWHM of the diffraction peak of the MC-PDGI/PAAm gels owing to a decrease in $\lambda_{A}$.

## 4. Discussion

The experimental results suggest that the swelling anisotropy (a pair of $\lambda_{L}$ and $\lambda_{D}$ at a given *Q*) of MC-PDGI/PAAm gels is governed by the elasticity of the PDGI bilayers, including buckling, whereas the *Q* itself is determined by both the PDGI bilayers and PAAm network. We attempt to explain these experimental results from a mechanical perspective. The Helmholtz energy density of the MC-PDGI/PAAm gel, *F*, is defined as the Helmholtz energy of the gel per unit volume in the as-prepared state. The equilibrium swelling state of the MC-PDGI/PAAm gel is where *F* reached its minimum value. We first apply the concept of an existing mechanical model that reproduces the anisotropic deformation behavior of a rectangular PDGI/PAAm gel [19]. Accordingly, *F* of a PDGI/PAAm gel is determined only from the following three contributions: polymer-solvent mixing $F_{\mathrm{mix}}$, elasticity of the PAAm network $F_{\mathrm{net}}$, and elasticity of the PDGI bilayers $F_{\mathrm{layer}}$. Other possible contributions, especially polymer–bilayer or solvent–bilayer interactions at the interfaces, were not considered. We assume no volume change occurring upon the mixing of the polymer or bilayer membrane of an MC-PDGI/PAAm gel with the solvent, perfect coupling of the gel and bilayer deformation, and fluidity of the PDGI bilayers. In this study, although the PDGI bilayer is thought to be viscoelastic [30], the viscous effect is negligible because the samples are in equilibrium swelling states, where they are mechanically relaxed. Instead of *F* itself, the difference in *F* between the state of interest and reference state, Δ*F*, is discussed. The prepared MC-PDGI/PAAm gel was used as the reference state.

According to the above assumptions, Δ*F* is simply the sum of three terms,

(2) $$\Delta F=\Delta F_{\mathrm{mix}}+\Delta F_{\mathrm{net}}+\Delta F_{\mathrm{layer}}.$$

For Δ*F*_mix_, we adopted the Flory–Huggins model for a polymer gel with a quasi-infinite molecular weight [31].

(3) $$\Delta F_{\mathrm{mix}}=\frac{\lambda_{L}\lambda_{D}{}^{2}}{v_{s}}k_{B}T\left(\left(1-\phi \right)\ln\left(1-\phi \right)+\chi \phi \left(1-\phi \right)\right)-F_{\mathrm{mix}}^{0},$$

where vs. is the volume of the solvent molecule; *k*_B_ is the Boltzmann constant; *T* is the absolute temperature (297 K); *ϕ* is the volume fraction of PAAm in the gel; and *χ* is the Flory interaction parameter between PAAm and water. $F_{\mathrm{mix}}^{0}$ is $F_{\mathrm{mix}}$ of the gel at the reference state. Since *ϕ* is a function of swelling ratio of the gel, Equation (3) can be also written as

(4) $$\Delta F_{\mathrm{mix}}=\frac{\lambda_{L}\lambda_{D}{}^{2}}{v_{s}}k_{B}T\left(\left(1-\frac{\phi_{0}}{\lambda_{L}\lambda_{D}{}^{2}}\right)\ln\left(1-\frac{\phi_{0}}{\lambda_{L}\lambda_{D}{}^{2}}\right)+\frac{\chi \phi_{0}}{\lambda_{L}\lambda_{D}{}^{2}}\left(1-\frac{\phi_{0}}{\lambda_{L}\lambda_{D}{}^{2}}\right)\right)\;-F_{\mathrm{mix}}^{0},$$

where *ϕ*_0_ is *ϕ* of the gel at the reference state.

Δ*F*_net_ is the strain energy density function of the PAAm network. As the deformation ratio of the PAAm network in the gel can be considered sufficiently small, we simply adopted the Neo-Hookean model, which reproduces the mechanical behaviors of a polymer network with small deformation [32].

(5) $$\Delta F_{\mathrm{net}}=\frac{G_{\mathrm{net}}}{2}\left(\lambda_{L}{}^{2}+2\lambda_{D}{}^{2}-3\right),\;$$

where *G*_net_ is the shear modulus of the PAAm network in the MC-PDGI/PAAm gel at the as-prepared state.

For Δ*F*_layer_, two cases should be considered. At *Q* > 1, the PDGI bilayers were stretched and expanded. Δ*F*_layer_ associated with the small-area expansion is expressed as follows [33]:

(6) $$\Delta F_{\mathrm{layer}}=\frac{\gamma_{\mathrm{ex}}}{d_{0}}\frac{\;{\left(\lambda_{L}\lambda_{D}-1\right)}^{2}}{\lambda_{L}\lambda_{D}}\;\;\;\;\left(Q>1\right),\;$$

where *γ*_ex_ is the area expansion modulus of the bilayers, and *d*_0_ is the average layer distance of the bilayers in the as-prepared gel. In contrast, at *Q* < 1, the PDGI bilayers were compressed and buckled. The energy of a bent lipid layer is generally expressed using the Helfrich model, which requires curvature of the lipid layers to calculate their bending energy [34]. However, meaningful estimation of the curvatures of the buckled PDGI bilayers in the shrunken MC-PDGI/PAAm gel is challenging at this stage. Therefore, instead of applying the Helfrich model to the experimental results, we adopted an approach to obtain a phenomenological formula based on the experimental results. We simply assume that the formula in Equation (6) is phenomenologically applicable, even in the case of $\lambda_{A}<1$.

(7) $$\Delta F_{\mathrm{layer}}=\frac{\gamma_{\mathrm{comp}}}{d_{0}}\frac{\;{\left(\lambda_{L}\lambda_{D}-1\right)}^{2}}{\lambda_{L}\lambda_{D}}\;\;\;\;\left(Q<1\right),\;$$

where *γ*_comp_ is the apparent area compression modulus of the bilayers.

The combination of Equations (4)–(7) leads to Equation (8), which is an equation of state of the MC-PDGI/PAAm gels.

(8) $$\Delta F=\frac{\lambda_{L}\lambda_{D}{}^{2}}{v_{s}}k_{B}T\left(\left(1-\frac{\phi_{0}}{\lambda_{L}\lambda_{D}{}^{2}}\right)\ln\left(1-\frac{\phi_{0}}{\lambda_{L}\lambda_{D}{}^{2}}\right)+\frac{\chi \phi_{0}}{\lambda_{L}\lambda_{D}{}^{2}}\left(1-\frac{\phi_{0}}{\lambda_{L}\lambda_{D}{}^{2}}\right)\right)-F_{\mathrm{mix}}^{0}\;+\frac{G_{\mathrm{net}}}{2}\left(\lambda_{L}{}^{2}+2\lambda_{D}{}^{2}-3\right)+\frac{\gamma }{d_{0}}\frac{\;{\left(\lambda_{L}\lambda_{D}-1\right)}^{2}}{\lambda_{L}\lambda_{D}},$$

where *γ = γ*_ex_ at *Q* > 1, and *γ = γ*_comp_ at *Q* < 1. vs. and *χ* for PAAm/water were estimated to be 3 × 10^−29^ m^3^ and 0.45, respectively [35]; *ϕ*_0_ was estimated to be 0.132 from the preparation conditions of the gel considering the density of dry PAAm; *d*_0_ was determined to be 105 nm based on the SAXD results; *G*_net_(*x*) was estimated to be 2, 7, and 12 kPa for *x* = 0.1, 0.3, and 0.5, respectively, from the tensile test of the rectangular PAAm(*x*) gels (see Figure S4); *γ*_ex_ and *γ*_comp_ were set as the fitting parameters.

**Figure 6:**
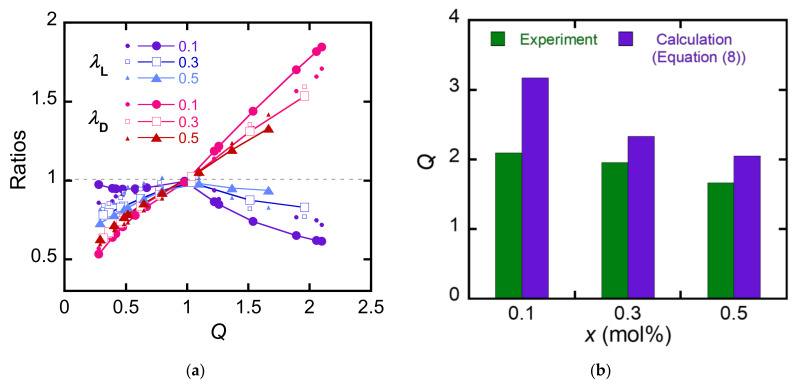
Estimation of the anisotropic swelling with Equation (8). (**a**) Experimental and estimated $\lambda_{L}$ and $\lambda_{D}$ of the MC-PDGI/PAAm(*x*) gels as functions of *Q*. The experimental values are shown with small symbols, while the estimated values are shown with large symbols. (**b**) Experimental and estimated *Q* of the MC-PDGI/PAAm(*x*) gels in pure water.

The validity of this equation can be evaluated using the following two points. First is the validity of $\lambda_{D}$ and $\lambda_{L}$ at given $Q=\lambda_{L}\lambda_{D}{}^{2}$. The MC-PDGI/PAAm gel at $Q=\lambda_{L}\lambda_{D}{}^{2}$ takes the pair of $\lambda_{L}$ and $\lambda_{D}$ that minimizes ΔF. The calculated $\lambda_{L}$ and $\lambda_{D}$ should match with the experimental values. Second is the equilibrium swelling behavior in water. The MC-PDGI/PAAm gel swollen in pure water takes $Q=\lambda_{L}\lambda_{D}{}^{2}$ that minimizes ΔF, which should match with the experimental *Q* in pure water. Figure 6a shows $\lambda_{D}$ and $\lambda_{L}$ at given *Q* estimated by Equation (7). For a comparison, the experimental $\lambda_{D}$ and $\lambda_{L}$ are also shown in the graph. We manually searched for a combination of *γ*_ex_, and *γ*_comp_ that minimize the residual sum of squares of the experimental and estimated values and chose *γ*_ex_ = 10 mJ/m^2^ and *γ*_comp_ = 0.4 mJ/m^2^ as the fitting parameters. The model reproduced the trend of swelling anisotropy of the MC-PDGI/PAAm gels, implying the suitability of the basic concept of this model. Although the swelling anisotropy of the MC-PDGI/PAAm gels does not depend on their crosslink density *x* experimentally, the estimated $\lambda_{D}$ and $\lambda_{L}$ at a given *Q* significantly depend on *x*. Therefore, a model wherein the elasticity of a polymer network affects the swelling anisotropy includes assumptions that differ from the real system. Figure 6b shows the estimated $Q=\lambda_{L}\lambda_{D}{}^{2}$ of the MC-PDGI/PAAm(*x*) gels swollen in pure water. The same fitting parameters were used for the calculation of *Q* with Equation (7). The estimated *Q* values were significantly larger than the experimental *Q* values, implying some problems in the model.

**Figure 7:**
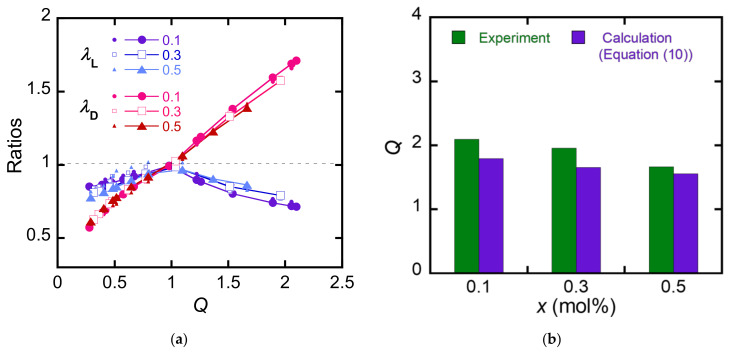
Estimation of the anisotropic swelling with Equation (10). (**a**) Experimental and estimated $\lambda_{L}$ and $\lambda_{D}$ of the MC-PDGI/PAAm(*x*) gels as functions of *Q*. The experimental values are shown with small symbols, while the estimated values are shown with large symbols. (**b**) Experimental and estimated *Q* of the MC-PDGI/PAAm(*x*) gels in pure water.

To solve the problem in Equation (8), the PDGI bilayer is considered to have not only the mechanical properties of a lipid membrane but also those of a rubbery membrane. Based on this modified assumption, Δ*F*_layer_ of the PDGI bilayers can be described as

(9) $$\Delta F_{\mathrm{layer}}=\frac{\gamma }{d_{0}}\frac{\;{\left(\lambda_{L}\lambda_{D}-1\right)}^{2}}{\lambda_{L}\lambda_{D}}+\frac{G_{\mathrm{layer}}d_{\mathrm{DGI}}}{2d_{0}}\left(\lambda_{L}{}^{2}+2\lambda_{D}{}^{2}-3\right),\;$$

where *G*_layer_ is the shear modulus of the PDGI bilayer, and *d*_DGI_ is the thickness of the PDGI bilayer, which was determined as 4.7 nm. The first term in Equation (9) represents the elasticity of the PDGI bilayer as a fluidic lipid membrane, and the second term represents the rubber elasticity of the bilayer. The corresponding equation of state of the MC-PDGI/PAAm gel is given by

(10) $$\begin{array}{cc} \Delta F=\frac{\lambda_{L}\lambda_{D}{}^{2}}{v_{s}}k_{B}T & \left(\left(1-\frac{\phi_{0}}{\lambda_{L}\lambda_{D}{}^{2}}\right)\ln\left(1-\frac{\phi_{0}}{\lambda_{L}\lambda_{D}{}^{2}}\right)+\frac{\chi \phi_{0}}{\lambda_{L}\lambda_{D}{}^{2}}\left(1-\frac{\phi_{0}}{\lambda_{L}\lambda_{D}{}^{2}}\right)\right)-F_{\mathrm{mix}}^{0}\\ & +\frac{1}{2}\left(G_{\mathrm{net}}+G_{\mathrm{layer}}\frac{d_{\mathrm{DGI}}}{d_{0}}\right)\left(\lambda_{L}{}^{2}+2\lambda_{D}{}^{2}-3\right)+\frac{\gamma }{d_{0}}\frac{\;{\left(\lambda_{L}\lambda_{D}-1\right)}^{2}}{\lambda_{L}\lambda_{D}}, \end{array}$$

where *γ = γ*_ex_ at *Q* > 1, and *γ = γ*_comp_ at *Q* < 1; *G*_layer_, *γ*_ex_, and *γ*_comp_ are set as the fitting parameters. Figure 7a shows $\lambda_{D}$ and $\lambda_{L}$ of the MC-PDGI/PAAm gels are estimated by Equation (10) as functions of *Q*. We chose *γ*_ex_ = 30 mJ/m^2^, *γ*_comp_ = 1.3 mJ/m^2^, and *G*_layer_ = 450 kPa as the fitting parameters by the manual searching of the optimum values that minimize the residual sum of squares. The calculated values nearly converge to one master curve, and these results are closer to the experimental results than those obtained using Equation (8), suggesting that Equation (10) represents a better model than Equation (8). A slight discrepancy between the experimental and calculated results was found at *Q* < 1; this is attributed to the too simple mechanical modeling of the buckled PDGI bilayer. A more complex mechanical model that considers the curvature of the membrane during buckling is necessary for more precise estimation. Figure 7b shows the estimated $Q=\lambda_{L}\lambda_{D}{}^{2}$ values of the MC-PDGI/PAAm(*x*) gels swollen in pure water. Although the estimated *Q* is slightly smaller than the real *Q*, the quality of the estimation using Equation (10) seems to be better than that using Equation (8).

Equation (10) effectively reproduces the experimental swelling results of the MC-PDGI/PAAm gels, suggesting that the PDGI bilayers have rubber elasticity in addition to lipid membrane elasticity. Two possible origins of PDGI rubber elasticity can be understood. One is the possibility that the PDGI bilayer itself has rubber elasticity; because PDGI is a polymerized lipid, the PDGI bilayers may have rubber elasticity owing to their characteristics as an assembly of mobile polymer chains such as elastomers. Second, the PAAm adsorbed onto the PDGI bilayer exhibits rubber elasticity because PDGI and PAAm interact with each other through hydrogen bonding; a thick adsorbed PAAm layer may be formed near the PDGI bilayer, which may behave as a composite membrane with both lipid and rubber elasticities [17].

Finally, the limitation of this study is described. First, the calculated $\lambda_{L}$ and $\lambda_{D}$ based on Equation (10) are slightly different from the experimental results in the de-swelling regime. This is because the elasticity of the PDGI bilayers in the de-swelling regime is not well described. To solve this problem, it is necessary to analyze the composite structure of the PDGI bilayers with absorbed PAAm and the structure of the buckled PDGI membrane upon de-swelling, which are technically challenging. Second, Equation (10) cannot be used as is to predict the equilibrium swelling state of MC-PDGI/PAAm gels PEG solutions. Swelling of the gels in PEG solutions should be estimated by taking into account the chemical potentials of water in the system including the PEG solution. Solving these difficulties for better understanding of mechanics of PDGI/PAAm gels is a subject for future works.

## 5. Conclusions

We have revealed the factors that control the super-anisotropic swelling of the MC-PDGI/PAAm gels. Swelling experiments showed that the swelling anisotropy of the MC-PDGI/PAAm gels was dominated by the elasticity of the PDGI bilayers. The SAXD results suggest buckling of the PDGI bilayers when compressed by de-swelling of the gels. The constructed equation of state of the MC-PDGI/PAAm gels, which can predict the anisotropic swelling behavior of MC-PDGI/PAAm gels, implied that the PDGI bilayer has elasticity as a lipid membrane as well as rubber elasticity.

The obtained model enabled the prediction of the anisotropic swelling behavior of a composite gel with a multi-cylindrical lamellar bilayer and polymer network, such as an MC-PDGI/PAAm gel, depending on its composition, concentration, cross-linking density of the polymer network, chemical species and density of the bilayer, and solvent species. For example, denser bilayers are expected to lead to a greater anisotropic swelling of the MC-PDGI/PAAm gel. Such investigations will enable the precise control of the anisotropic swelling behavior of bilayer/polymer composite gels and creation of gel actuators with controlled swelling anisotropy.

## Data Availability

The data presented in this study are available from the corresponding author upon request.

## Supplementary Materials

The following supporting information can be downloaded at: https://www.mdpi.com/article/10.3390/polym15071624/s1, Figure S1: 2-D X-ray diffraction image of the MC-PDGI/PAAm(0.1) gel swollen in PEG aqueous solutions, Figure S2: FWHM of the first-order X-ray diffraction peak of the MC-PDGI/PAAm(0.1) gel in the swelling regime, measured with longer camera length. Figure S3: swelling of the string PAAm(0.1) gels in PEG aqueous solutions. Figure S4: stress-strain curves of the rectangular PAAm(*x*) gels. Table S1: List of raw materials with their supplier and assay.
