# Supplementary material for: Mechanical Model for Super-Anisotropic Swelling of the Multi-Cylindrical PDGI/PAAm Gels
Source: Polymers (Basel). 2023 Mar 24;15(7):1624. doi: 10.3390/polym15071624 (PMC10096863; doi:10.3390/polym15071624)
Supplement: Supplementary file 1 [file polymers-15-01624-s001.zip › polymers-2258049-supplementary.docx]

Supplementary Materials for

Mechanical model for super-anisotropic swelling of the multi-cylindrical PDGI/PAAm gels

Tasuku Nakajima ^1,2,^*, Kei Mito ^3^, and Jian Ping Gong ^1,2^

^1^ Faculty of Advanced Life Science, Hokkaido University, N21W11, Kita-ku, Sapporo 001-0021, Japan

^2^ Institute for Chemical Reaction Design and Discovery (WPI-ICReDD), Hokkaido University, N21W10, Kita-ku, Sapporo 001-0021, Japan

^3^ Graduate School of Life Science, Hokkaido University, N10W8, Kita-ku, Sapporo 060-0810, Japan

***** Correspondence: tasuku@sci.hokudai.ac.jp


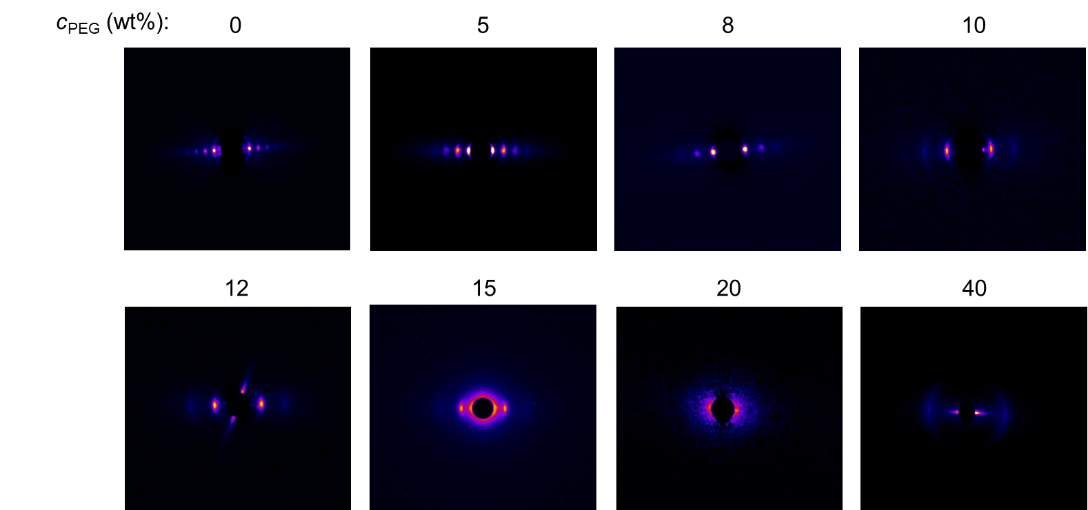


**Figure S1**. 2-D X-ray diffraction image of the MC-PDGI/PAAm(0.1) gel swollen in PEG aqueous solutions.

**Figure S2**. FWHM of the first-order X-ray diffraction peak of the MC-PDGI/PAAm(0.1) gel in the swelling regime, measured with longer camera length.

**Figure S3**. $\lambda_{L}$ and $\lambda_{D}$ of the string PAAm(0.1) gels as functions of *Q*. Reproduced from the data shown in ref. 18 (K. Mito *et al.*, *Polymer* 2017, 128, 373–378), Copyright 2017, with permission from Elsevier.

**Figure S4**. Uniaxial stress-strain curves of the rectangular PAAm(*x*) gels at their reference state, where *x*=0.1, 0.3 and 0.5. The gels were cut into the dumbbell shape (gauge length: 12 mm, width: 2 mm) and tested. Strain rate was 0.14 s^-1^. Young’s modulus of the gel was determined as initial slope of the curve. *G*_net_(*x*) was calculated by dividing the Young’s modulus by 3.

|  | Supplier | Assay |
| --- | --- | --- |
| Acrylamide | Junsei Chemicals | 98%+ |
| *N,N*'-Methylenebisacrylamide | Wako Pure Chemical Industries | 99%+ |
| Irgacure 2959 | BASF SE | 99% |
| Sodium dodecyl sulfate | MP Biomedicals | 99%+ |
| Polyethylene glycol (Mn: 21,170) | Wako Pure Chemical Industries | ~100% |
| Itaconic acid anhydride | Sigma Aldrich | 95%+ |
| Dodecanol | Wako Pure Chemical Industries | 95%+ |
| Glycidol | Wako Pure Chemical Industries | 90%+ |
| Pyridinium *p*–toluenesulfonate | Wako Pure Chemical Industries | 97-102% (by titration) |

**Table S1**. List of the chemicals used in this study with their supplier and standard assay.
